# Creating a safe space: medical students’ perspectives on using actor simulations for learning communication skills

**DOI:** 10.1186/s12909-024-06184-6

**Published:** 2024-10-28

**Authors:** Asta Kristiina Antila, Sari Lindblom, Pekka Louhiala, Eeva Pyörälä

**Affiliations:** 1 Department of Public Health, Clinicum, Faculty of Medicine and Department of General Practice and Primary Health Care, University of Helsinki, University of Helsinki and Helsinki University Hospital, Helsinki, Finland; 2 University of Helsinki, Helsinki, Finland; 3 Health Sciences Unit, Faculty of Social Sciences, and Department of Public Health, Clinicum, Faculty of Medicine, Tampere University, University of Helsinki, Tampere, Helsinki, Finland; 4 Centre for University Teaching and Learning, Faculty of Educational Sciences, University of Helsinki, Helsinki, Finland; 5 Department of Public Health, University of Helsinki, Helsinki, PO Box 20, Finland

**Keywords:** Medical students, Doctor-patient communication, Communication skills studies, Psychological safety, Feedback, Simulations, simulated patients, Actors as patients, Content analysis

## Abstract

**Background:**

Communication skills are an essential part of clinical competence that need to be acquired during health professions education. Simulations are extensively used for learning communication skills and have long been integral to medical degree programmes. In this research we use qualitative methodology to explore fourth-year medical students’ experiences in simulations aimed at improving versatile doctor-patient communication, focusing on their learning with trained actors.

**Methods:**

The data comprises reflective writings from 208 fourth-year medical students, gathered after a communication skills course. These students provided informed consent for their writings to be included in the research. We performed an inductive qualitative content analysis on the textual data, with findings presented as themes, supported by categories, codes, and excerpts from raw data to enhance the trustworthiness of the analysis.

**Results:**

We identified eight key themes capturing students’ learning experiences through simulations: practising in a safe learning environment, valuing feedback, gaining new perspectives, finding simulations valuable and enjoyable, boosting confidence and self-knowledge, and viewing simulations as authentic and engaging learning opportunities. Some students offered critical perspectives on simulations. Throughout the course, students learned diverse aspects of patient care, emotional and behavioural communication dynamics, and lessons from medical errors. Some students offered critical perspectives on simulations, and a few indicated they did not learn anything new.

**Conclusions:**

A safe learning environment is vital for encouraging learners to explore, make errors, and absorb feedback to improve their communication with patients. Students predominantly valued the communication skills training with actors and the constructive feedback received and given in the debriefing discussions. However, some students expressed critical views toward simulations. Simulations are not static; they evolve and require continual improvements. Hence, we advocate for the ongoing exploration and enhancement of communication skills learning methods, including simulations, with careful consideration for students’ vulnerability and the importance of psychological safety. Additionally, it is critical to address students’ perceptions that certain clinical teachers prioritise biomedical knowledge over communication skills. Providing tailored training for teachers regarding the learning methods and the desired outcomes of communication courses is essential.

## Background

Sir William Osler, one of the pioneers of modern clinical training famously stated, “The good physician treats the disease; the great physician treats the patient who has the disease” [1, p. 665]. Breaking life-changing news to a patient is a challenging task that requires a combination of clinical skills, communication, knowledge, problem-solving, and possibly physical examination. If communication falters, the effectiveness of these other skills diminishes [2].

Effective doctor-patient communication influences patient outcomes [2–8]. It fosters mutual understanding, patient satisfaction, addressing the need to be seen and heard. It actively engages patients in their treatment, supports their emotional well-being and lays a foundation for a strong doctor-patient relationship, correlating with increased satisfaction and commitment to care [4]. Mistiaen et al. [3] suggest that empathetic communication can alleviate patients’ perceived pain. Conversely, poor communication poses significant risks, leading to adverse events and claims [2]. Proficient communication not only benefits patients but also enhances healthcare professionals’ well-being and overall healthcare quality [4, 9, 10].

Communication skills, although complex, can be learnt, and various learning methods have been developed for this in health professions education. Kurtz et al. [2] suggest that experiential learning methods are best suited for teaching these skills. Koponen et al. [11] compared simulated patients, role-plays, and theatre in education, concluding all methods are suitable. Simulations are particularly effective to address breaking bad news and emotional aspects of patient encounters [12, 13]. They can be organised with peers, laypersons, or professional actors portraying patients [11, 13–15]. Previous research [14, 15] highlights that the improvisational skills of professional actors add significant value by providing a more realistic experience and challenging students to step outside their comfort zones, all within a safe learning environment for both students and patients.

Simulation sessions feature realistic patient scenarios followed by feedback discussions that facilitate reflection [16]. These discussions emphasize key communication elements, including presence, listening, understanding the patient’s perspective, reciprocity as well as emotions as a crucial aspect of interpersonal communication [17]. Krishnasamy et al. [17] suggest that guided reflection aids students in contemplating patient encounters, empathy, and compassion.

Simulations stand as a widely adopted learning method in communication skills training to medical students globally. Since 1994, simulations have been a core component of communication skills courses in Finnish medical education. Our previous research delved into students’ emotional reflections on delivering bad news [18]. This study aims at examining medical students’ experiences of learning communication skills through actor simulations. It also explores students’ perceptions of the learning outcomes from these simulation-based courses. This research seeks to enhance our understanding of simulations as an educational approach and identify potential areas for further development.

The specific research questions are:

1. How do the medical students describe their experiences of learning communication skills in simulations with actors portraying patients?
2. What insights do the students articulate of their learning outcomes from the communication skills course using simulated patients?

## Methods

### Study context

The Finnish undergraduate medical education spans six years, totalling 360 European Credit Transfer and Accumulations System (ECTS) credits, with a primary goal of preparing graduates for medical practice in primary health care. An integral part of the curriculum is communication skills training, which incorporates both real patient encounters and simulations involving professional actors.

This study explores a communication skills course for the fourth-year students where the scope extends beyond routine scenarios to encompass a diverse range of patient interactions, including intricate situations. This involves fostering patient engagement in shared decision-making, addressing challenges with unmotivated patients, managing patient fears and resistance to treatment plans, handling medical errors, and breaking bad news.

The simulation involves a small group of six to ten students, an actor, and a clinical teacher. Students take turns playing the role of the doctor, while the rest of the group observes. The actor improvises scenarios based on short vignettes. Following each simulation, a reflective debriefing discussion, facilitated by the clinical teacher, allows participants to share observations, provide feedback, and collaboratively explore communication strategies. The discussion starts with the perspectives of the student doctor, followed by the actor, and then involve the entire group. Learning in these simulations is experiential, enabling students to actively participate, observe, provide feedback, and reflect on communication, fostering a comprehensive learning experience.

### Data

A total of 208 fourth-year medical students from two consecutive cohorts in 2014 and 2015 participated in the study. After the communication skills course, all the medical students in the course completed an online survey consisting of three distinctive components, each generating a different type of data: first, they reflected on a challenging patient scenario from a physician’s perspective; second, they rated their communication skills with statements using 5-point Likert scale; and third, they responded in writing to an open-ended question, describing in their own words their experiences with the course’s learning methods. This study focuses on the qualitative data derived from the students’ written responses to the open-ended question about the course’s learning methods, which were explored using content analysis. Research on the patient scenario has been published previously [18], and the results of the Likert-scale self-assessment will be presented in a separate publication that employs statistical methods.

Communication skills courses in our unit are today conducted in the same manner as they were in 2014 and 2015. Thus, the material remains relevant to the current landscape of medical education.

Before collecting the data, we presented the study to the Undergraduate Medical Education Planning Committee and received permission to proceed. The research adhered to the ethical standards outlined in the Declaration of Helsinki and followed the guidelines established by the Finnish National Advisory Board on Research Integrity [19, 20]. We informed the students about the study and participation was voluntary. All respondents gave informed consent to use their answers in the study.

### Data analysis

The research is qualitative in nature. We employed qualitative content analysis to examine responses to open-ended questions regarding simulation as a learning method, using two datasets from consecutive student cohorts. While content analysis has traditionally served as a research method for quantitatively processing text data, this study adopted the qualitative content analysis outlined by Elo and Kyngäs and Elo et al. [21, 22]. As described by Short et al. [23, p. 416], qualitative content analysis is well-suited for extracting meaning from individuals’ written reflections, capturing “a rhetoric presented in words and narrative texts”. They underscore the method’s effectiveness in building understanding “by the use of words, phrases, and language” [23, p. 416].

The data consisted of students’ texts, ranging from individual words to phrases up to five sentences in length, with the majority composed of three to four sentences. In the preparatory phase [21, 22], the two authors (AA and EP) thoroughly read the material. The writings were coded for similarities, sample quotations were extracted, and preliminary categories were formed, discussed, and finalised. The frequency of the categories was calculated. Themes were then derived from these categories, encapsulating key aspects of students’ views on using simulations with professional actors to learn communication skills. This is summarised in Tables 1 and 2. To ensure systematic and flexible coding and analysis, AA utilized computer-aided analysis through Atlas.ti [23].

**Table 1:** Students’ experiences of learning communication skills in simulations with actors as patients

Themes	Categories (No. of quotations)	Codes	Sample quotations
**1. Practising in safe learning environment**	Good to observe others (51)	Observing peers, seeing others acting in different encounters with different patients, different personalities, noticing both good and bad approaches, illustrative	“By observing you learn more than just by acting yourself”
	Prepares for challenging encounters (34)	Even once encountering challenging patient, preparing for interview with difficult patients, more familiar when in real life, glimpse of the idea of BBN, how to approach	“Practising with an actor is important, just that even once you somehow get to try the situation before you end up in serious action.”
	Learning by doing (31)	Doing, conducting consultation, practical training, exploring	“…practical training and doing is ultimately how these skills are learned and it is important not to practice them for the first time with real patients”
	Safe environment (11)	Safe environment for experiences, safe to make mistakes, safe surrounding, supportive environment, and atmosphere	“An opportunity to get to experience feelings that you will surely confront in real life as well in a safe environment is unique”
	Learning from errors (3)	Mistakes as a source of learning, safe to make in the context of studies, how to proceed with errors	“From every mistake, be it a friend’s or your own, you always learn. So you should mess up really hard during the studies not to make the same mistakes in real life.”
**2. Getting feedback is crucial**	Corrective and constructive feedback is crucial (36)	Feedback should be straight, constructive, corrective, concrete, feeding forward, ideas for improvement	“The feedback could have been sharper. Failures and arrogant attitudes should not be swept under the carpet.”
	Feedback from others is important (22)	Feedback is especially important, others’ suggestions useful, confirming own ideas, gives information of competence	“You can feel what was more or less successful, but feedback is though very important”
	Feels good to get feedback (21)	Feels good/nice to get feedback, it is nice to hear opinions of others	“It feels good to get feedback”
	Actors’ feedback is important (19)	Feedback from actors was of exceptional value, specifically feedback of how the needs of the patient were met, the most teaching thing was the feedback from actors	“Actor’s comments, how the patient experienced the situation, where the most important”
	Learning to give and receive constructive feedback (11)	Challenging to give corrective feedback, giving feedback to a friend, not easy to give, receiving feedback, not easy to adjust with the feedback	“You learn to give feedback in the process, constructive feedback”
	Positive feedback is appreciated (9)	Positive feedback is important, appreciated, good to bring forth students´ strengths	“The feedback given was mostly positive, which was good”
**3. Gaining new perspectives**	New perspectives through the analysis (44)	Analysing immediately after, brings new/wider perspectives, in discussions things you do not notice yourself, interesting to hear the different ideas/interpretations, good to hear other opinions, debriefing thoughts the case provoked, putting experience in words, thought provoking	“Going through the things and analysing the situation right away gave a bunch of new perspectives and skills to outline the situation plus both the doctor’s and the patient’s world and emotional experiences.
	Reflection (25)	Thinking the cases through, how to respond in situations, reflect own thoughts and behaviour, what to do differently, making mental models/schemas of the codes of conduct, opening ideas	“Reflecting own behaviour is useful”
	Discussing the case with the group is useful (25)	It was nice to discuss, necessary, useful in finding solutions, good to discuss, rewarding	” It was funny to notice how different things people had noticed, but on the other hand, everyone valued the same things, i.e., looking into the eyes, compassion, respecting others, listening to the patient.”
	Clinician’s perspective (11)	Good to hear clinicians’ reflections on authentic patient encounters, own experiences and practical tips, suggestions for alternative ways to act, medical aspects to the cases	“it was good to hear clinicians’ perspectives on how to act in real patient encounters”
	Debriefing is important (8)	Debriefing the simulation for learning, were good, important to get to debrief the thoughts, good to ventilate	“This was the best part. Debriefing with teachers who were good and stressed the right things.”
**4. Simulations are useful and fun**	Useful (55)	Very teaching, particularly good, useful, best part, excellent/good practice, useful teaching method, good examples	“The simulations were very instructive”
	Most instructive (12)	The most teaching, where learning occurs, most rewarding	“The most rewarding part.”
	Simulations and debriefings seamlessly together (6)	Seamlessly together, feedback sessions are important part of simulations	“Debriefing sessions with feedback discussions are important part of simulations”
	Funny experience (3)	Funny experience, very funny	“It was a funny experience.”
**5. Critical perspectives**	Lack of authenticity (19)	Simulations are not realistic due to the observation, unreal and weird, real patients more authentic, hard to think as real, unauthentic, artificial, useless sentimentality, felt unnatural, hard to take seriously	“Always a bit artificial having to play the theatre”
	Should not concentrate on medicine. (7)	Criticising medical knowledge is detrimental and should be avoided, unnecessary concentration on medicine, should not concentrate on medicine, lack of clinical ability irritates	“The medical knowledge of the students should not be judged in the practices.”
	Heavy experience (6)	Long feedback discussions were uncomfortable, hard to change behaviour and needs repletion, acting in panic, heavy experience, difficult to act	“I believe this part was very heavy for many students.”
	Nothing new (6)	Nothing new, felt everything was already familiar	“In and of myself nothing new”
	Too little time (3)	Useful but too little time, short feedback sessions	“Useful as such but there was too little time used on these”
	Self-criticism (2)	Too sensitive, too unorganised, lack of clinical ability irritates	“Made me feel I am too sensitive and unorganised for a doctor”
	More intensity in practices (1)	Only challenging consultations	“These practices should be more intense. We should not waste time on basic consultations but use only difficult cases: discussing errors, breaking bad news, using interpreters with immigrant patients, angry patients and so forth”
**6. Building confidence**	Self-confidence (24)	Affirmation, confidence, confronting insecurity, encouragement.	“I got a patient who was shouting and roaring, with whom I could not get any kind of contact. But with this experience, I learned that I could do this. ϑ”
	It went quite well (6)	Went quite well, ok, feeling good	” I got a good feeling about how it went.”
	Getting familiar with physician’s professional role (5)	Physicians’ role to help, finding one’s own role, physicians’ authority.	“I managed to create a reliable and knowledgeable impression even though the subject was not too familiar, and it was partly difficult to answer the questions asked by the patient.”
	Encounters succeed with peaceful and independent attitude (5)	Independent and peaceful attitude and well succeeded practice, being peaceful and systematic, relying on own tranquillity	“Tranquillity and being systematic work in almost all situations”
**7. Enhancing self-knowledge**	Self-knowledge (15)	Own feelings, reactions, and behaviour in different situations	“Mostly I learned self-knowledge, how I react in unexpected situations (my patient was trying to leave in midstream).”
	Seeing yourself in the eyes of others (13)	How others see me and my behaviour, how others think about me, how you appear, uncertainty and stress may not be seen	“Although I feared that my speech was slow or delayed, others did not notice it.”
	Recognising one’s own strengths and weaknesses (5)	Noticing own strengths and weaknesses, what has one did right and wrong, what went well	“You notice your own weaknesses and strengths in a different way than if you were just trying to judge them yourself.”
**8. Encountering authentic and exciting experiences**	Authentic and exiting situation, feelings genuinely present (8)	Authentic discussion with actor-patient was exiting, scary but rewarding, genuine feelings	“Actors were genuine, so my own thoughts and feelings came out as genuine”
	Good to discuss with the actor (8)	Get to talk to actor patient, actors present a layman’s perspective, actors’ thoughts and feelings, patient’s perspective	“It was especially rewarding to be able to ask the actor about the patient’s feelings and thoughts afterwards”

**Table 2:** What the students learnt in simulations

Themes	Categories (No. of quotations)	Codes	Sample quotations
**1. Patient-related issues**	Meeting the patient’s feelings (29)	No need to fear the patients’ feelings, putting up with unpredictable reactions of the patient, giving time for calming, giving space, being responsive, noticing the emotions, facing the emotions of the patient	“I learnt the patient is not always reacting the way you expect. It takes time for emotional reaction to develop”
	Encountering different patients (12)	Different types of patients, persons	“There are may kinds of patients”
	Acting upon the patient’s needs (11)	Concentrating on the things the patient worries about, acting based on patient’s reactions, giving time, informing the patient with their level of understanding,	“Nothing is self evident in the consultation. The informing of the patient must be in their level f knowledge and understanding”
	Motivating the patient (9)	Find a way to motivate, motivating	“I learnt to motivate a challenging patient”
	Listening to the patient (6)	Listening	“You need to listen carefully what the patient says”
	Having respect for the patient (5)	Respect, do not judge	“I learnt the patient needs to be respected and treated appropriately in every situation”
	Ensuring the continuity of the treatment (3)	Ensuring the continuity, written/clear treatment plan	“In challenging encounters especially, there needs to be a follow up plan to build trust and safety”
	Collaborating with the patient (1)	Planning and following the treatment together	“e.g., supporting the weight losing, you can make the plan together and then see how it works on the next consultation”
	Acknowledging the patient’s situation holistically (1)	Patient’s situation holistically	“Focusing on certain details that are not related to the patient himself, such as family etc”
**2. Behavioural issues**	Practical tips for challenging situations (26)	practical tips, found the tools to BBN in the small group, different techniques, ways to act, concrete, pragmatic tips,	“I got more practical tips for BBN”
	Understandable communication (17)	Language, clarity, directness, sharing information, explaining carefully, focusing on the most critical issues, using common language, using written material to support the message	“It’s very important to be able to explain to the patient what it’s all about”
	Not just one way to act but more (16)	Different ways encountering, diverse ways doing things	“I learnt we have many different ways encountering challenging situations”
	Taking the doctor’s role (9)	Being a doctor, professional role, taking the doctor’s role	learned to take the role of a doctor and a professional
	Preparing for consultations (7)	Preparing for the case, for puzzling questions in advance,	“Difficult questions like about percentages should be prepared in advance”
	Encountering patients is not all about medicine (2)	More than medicine	“A great deal about successful doctor-patient encountering is not about medicine “
	Basic skills in communication (2)	Basic skills, interacting with the patient	“I learned patience and interacting with the patient”
	Looking the patient in the eyes (1)	Looking in the eyes	” All seemed to respect the same kind of things like looking in the eyes.”
	Talking without saying anything (1)	Talking	“How to talk for a long time without saying anything”
**3. Emotional issues**	Own feelings and reactions in challenging situations (23)	Idea of how one feels, behaves, and reacts, how situation affects, how to act, self-knowledge,	“Got at least a small glimpse of how it feels to break bad news to a patient” “Self-knowledge, how I react to unexpected situations”
	Compassion/empathy (9)	Challenge of empathy, enough of empathy, patients’ perspective, compassion	“Learnt about the challenge to be empathetic but not take the patient’s fate too much on own shoulders” “All situations can be overcome if you have enough empathy”
	Tolerance of silent moments (9)	Tolerance of silence, silence is important, quiet moments can be longer	“Silence is also an important part of the consultation; you shouldn’t rush the patient out”
	Tolerance of uncertainty (8)	Tolerance of uncertainty, ok to feel uncertain, preparing to meet anyone, uncertainty will not necessarily show, the physician need not to know everything, you cannot’ always prepare yourself	“The patient/actor may not even notice your uncertainty and tension” “It’s ok to say you don’t know”
	Calming the atmosphere (8)	Concentrating, do not rush, patience, creating a calm encounter, staying calm, calming the situation	“Being patient is usually a good way of acting” “Creating a calm encounter”
	Building trust (2)	Building trust	Building trust (2)
**4. Learning from errors**	Learning from errors and dealing with them (19)	Errors might be done – important to apologise, admitting the errors, thinking what to do in case of errors, facing errors	“The meaning of admitting own mistakes and apologising was highlighted in a specific way in a few cases”
**5. Nothing new**	Things that were already familiar (6)	Nothing really new, did not teach me anything, not much	“I felt that I already knew the all the things that came up”

Initial findings from the study were shared at the AMEE (the International Association for Health Professions Education) conferences in 2019 and 2021, where we received valuable feedback for the precision of the study’s findings. In the results section, we outline the detailed progression of the analysis, from raw data to meaning units, coding, categorization, and themes, enhancing transparency and supporting the study’s rigor, as recommended by Erlingsson and Brysiewicz [24].

## Results

This study centres on student perceptions regarding simulations and subsequent debriefing discussions. Out of 227 students, 208 (92%) took part in the study. 19 students declined to participate, and consequently, their answers were excluded from the dataset.

### Students’ experiences of learning communication skills in simulations with actors as patients

The study explored students’ experiences while learning communication skills through simulated scenarios with professional actors portraying patients. Using content analysis, we identified eight overarching themes that encapsulate these experiences. Table 1 provides an overview of these themes, including categories, codes, and illustrative data samples. These themes highlight what students found significant during the simulations and subsequent feedback sessions. Our findings reveal both positive experiences associated with simulations and, critical reflections that shed light on the challenges inherent in this type of experiential learning.

The students’ experiences regarding learning communication skills in simulations revolved around eight themes: practising within a safe learning environment, getting feedback is crucial, gaining new perspectives, simulations are useful and fun, critical perspectives, building confidence, enhancing self-knowledge, and encountering authentic and exiting experiences. Further elucidation of these themes follows in the subsequent description.

The students described most frequently simulations as creating a safe learning environment for practising doctor-patient communication. This setting allowed them to gain experience handling diverse patient interactions, actively engage in simulated consultations and observe. Simulations created a safe space for exploring doctor-patient communication and learning from potential communication errors and setbacks without causing real harm to patients, thereby effectively preparing students for real-life patient consultations.

Students emphasised the crucial role of feedback following simulation practices in their learning process. They expressed a desire for honest, constructive feedback that helped them improve their communication skills. Even when their actions did not require correction, they appreciated suggestions for alternative communication approaches. Many students wanted more of this type of feedback during reflective debriefing sessions. Such feedback enriched their perspectives and provided deeper insights into doctor-patient interactions. The feedback from professional actors, offering the patients’ viewpoint, was highly valued.

Simulations also served as a platform for students to learn how to give and receive feedback. However, they found it challenging to provide constructive or corrective feedback to friends, as they wanted to avoid causing discomfort. They also acknowledged the difficulty in adjusting to feedback received. Despite these challenges, students valued the feedback and appreciated assessments of their communication skills. Overall, they considered feedback an indispensable component of the simulations.

Students perceived simulations as opportunities to gain new perspectives, particularly through analysing and discussing cases in reflective feedback sessions immediately after the exercises. They valued the insights provided by clinician teachers, who shared reflections on real patient encounters. Engaging in small group discussions following the cases was not only enjoyable for students but also crucial in addressing communication-related challenges. The intensity of the simulations encouraged students to reflect on the cases and debrief the thoughts evoked by the exercises. These reflections included discussions about their reactions, and exploring alternative approaches, which stimulated new ideas.

The theme that described simulations as both useful and fun captured students’ experiences, portraying simulations as the most engaging and informative method for learning communication skills. This positive disposition reflected students’ views of simulations as an effective learning method. They found simulations both rewarding and useful, with the reflective debriefing sessions complementing the learning process seamlessly.

Engaging in simulations allowed students to gain confidence in their professional communication skills. Overall, they expressed satisfaction with the simulated consultations and felt a sense of accomplishment. Their self-efficacy improved with recognition and encouragement, which supported their future endeavours, and their ability to manage uncertainties. Students faced exhilarating situations with a composed and independent demeanour, learning to navigate professional roles more effectively through these practices.

Students expressed critical perspectives regarding simulations, perceiving the practices as unrealistic and artificial. They voiced discomfort with role-playing and criticised the emphasis on communication skills rather than medical content. Additionally, the intensity and length of simulations and debriefings caused stress and discomfort for some students, hindering behavioural changes. Views on session duration varied, with some students finding them too long or too short. There were also differing opinions on the allocation of time between simulations and feedback. One area of criticism concerned the difficulty of patient scenarios. Some students felt that the exercises lacked novelty, while others wished for more challenging cases to enhance their practice.

During reflective debriefing sessions, students acknowledged that simulations enhanced their self-knowledge by revealing how others perceived their consultations. They recognized that much of the excitement and stress they experienced during simulations was not noticeable for observers. Additionally, reflecting on their own emotions and reactions to these encounters contributed to a deeper understanding of themselves, better preparing them for future interactions. In these scenarios, students identified their strengths and weaknesses, gaining insights into what worked well and areas for improvement.

Students described simulations with actors portraying patients as both authentic and exciting experiences. They felt that the actors played their roles realistically, eliciting genuine emotions and reactions from the students.

### What the students learned in simulations

The students’ reflections on their learning from simulations revealed a depth of knowledge that surpassed the specific learning outcomes of the course, primarily addressing various facets of communication competence. In analysing students’ responses (Table 2), we identified five key themes encompassing learning outcomes expressed by students: patient-related issues, behavioural aspects, emotional aspects, and learning to deal with errors. Additionally, we recognised the perception of not learning anything new during the course.

The theme involving issues related to patient care encapsulated the students’ reflections on prioritising patient-centeredness in their interactions. This theme encompasses categories such as learning to encounter and collaborate with diverse patients, adeptly listening to and motivating patients, ensuring continuity of treatment, and addressing patients’ needs and emotions. Moreover, it included showing respect for patients and understanding their situations holistically, including the support offered by their families and significant others.

The theme of behavioural aspects covered students’ accounts of acting as a professional when interacting with patients. This theme was constructed based on categories such as acquiring practical guidance for handling challenging situations, employing clear and comprehensible communication, and exploring different approaches to behaviour during patient encounters. This includes, taking on the doctor’s role, preparing for consultations, and actively engaging in them.

Within simulations, students gained insights into the emotional aspects of patient encounters. They had the opportunity to cultivate and develop empathy and compassion while caring for patients, navigating moments of uncertainty and silence during consultations, building trust, and fostering a calm atmosphere. Additionally, students enhanced their self-knowledge by understanding their own reactions and emotions in challenging situations.

Simulations provided a secure environment for students to learn how to deal with errors.

However, some students expressed that the simulations did not offer them anything new, indicating that they were already familiar with the issues covered.

## Discussion

This study aimed to deepen our understanding of medical students’ experiences of learning communication skills through simulations with actors portraying patients. Students’ written reflections highlighted the value they placed on simulations, emphasising the benefits of practising in safe, realistic environment, receiving feedback, gaining new perspectives, enjoying the practice, and enhancing self-confidence, and self-knowledge. However, some students were reluctant to fully engage and expressed critical viewpoints.

Psychological safety, as defined by Edmondson [25, 26], emerged as crucial. It involves feeling secure to take interpersonal risks, speak up, and express oneself authentically, and admit mistakes [25–27]. In our courses, students faced tasks like breaking bad news and disclosing medical errors. The safe environment allowed them to make mistakes without risking the patient care, thus preparing them for real-life scenarios. This psychological safety enabled learners to be fully present in the moment and focus on the patient [28]. Such preparation is expected to reduce anxiety and stress in authentic situations, leading to more effective interactions, better patient care, and improved outcomes. One of the key takeaways from this study is the importance of all faculty appreciating and enhancing the psychological safety in learning environments to support this progress.

Actor simulations provided a supportive environment for developing communication skills, aligning with previous research [29, 30]. Feedback from actors, peers, and clinical teachers was essential for identifying strengths and areas for improvement [30–32], particularly concerning the emotional aspects of consultations [32]. Feedback from clinical teachers, especially when specific and actionable, reinforced experiential learning [33] and was most effective when delivered by trusted individuals [34].

The process of giving and receiving feedback was itself a valuable learning experience. Peer feedback in collaborative context was instrumental in shaping professional behaviour and learning to provide feedback, a skill often underemphasised in formal curricula [35]. Students found the simulations enjoyable and beneficial, appreciating the opportunity to analyse scenarios and reflect collaboratively, which enhanced their confidence and self-knowledge [36, 37].

Despite the benefits, some students found simulations exhausting and too focused on biomedicine rather than communication. Addressing these criticisms is vital, including training teachers to emphasise communication skills in simulations instead of focusing on clinical aspects. Previous research highlighted the complementary roles of real and simulated patients, with simulations being more effective for communication training [38]. While simulations can feel artificial, their emotional impact is real, helping students manage future professional challenges [39–41]. Ultimately, engaging with actor-patients allowed students to contemplate patient perspectives, reflect, recognize behaviours and emotions, and deal with errors, though some felt they gained no new insights, possibly due to similar prior experiences.

### Limitations of the study

One limitation of the study is that the data was collected in 2014 and 2015. However, the simulation training, including the patient cases, has remained the same. Thus, the research context has not changed, and the results are still relevant and important for the development of communication skills courses.

We employed qualitative content analysis to develop themes and categories [20–23], ensuring the trustworthiness by detailing our data collection, coding and participant characteristics [21]. To enhance credibility [21], we presented themes, categories, codes, and citations, refining results through researcher discussions and educator feedback. The study’s strength lay in the diverse perspectives of the authors, offering both insider and outsider viewpoints. AA, an external researcher, was familiar in the communication program and has been working within medical education since 2007. EP responsible for the communication skills studies over 25 years, delivered the introductory lecture, professor of medical philosophy and ethics PL served as a facilitator in simulations, and professor of higher education SL contributed as an external researcher. Reflections from 208 students allowed comprehensive analysis, though the small refusal rate (19 out of 227) leaves the impact of missing contributions unclear. We included divergent opinions to provide a balanced perspective. Using mandatory assignments as data provided extensive insights but also elicited critical viewpoints on the exercises’ authenticity and feedback focus, highlighting areas for improvement.

## Conclusions

Simulations in medical education are a powerful tool for enhancing communication skills. This qualitative study explored students’ experiences with actor-based simulations to understand this educational method from their perspective. Findings highlight the importance of simulated patients in providing realistic learning opportunities for handling difficult patient scenarios. A psychologically safe environment is crucial for building confidence, encouraging reflection, and fostering feedback skills, all essential for their future healthcare roles. While beneficial, some students resisted simulations or noted sessions focused too much on biomedicine rather than communication. Addressing these issues is essential for optimising simulations in medical education.

## Data Availability

The research data are in Finnish and available from the corresponding author.
